# Draft genome sequence data of *Colletotrichum siamense* isolated from *Camellia japonica* in the United States

**DOI:** 10.1016/j.dib.2025.112349

**Published:** 2025-12-07

**Authors:** Kenneth R. Leep, Renee S. Arias, Warren E. Copes, Siva P. Kumpatla

**Affiliations:** a United States Department of Agriculture (USDA), Agricultural Research Service (ARS), Thad Cochran Southern Horticultural Laboratory (TCSHL), 810 Hwy 26 W, Poplarville, MS, USA; b United States Department of Agriculture (USDA), Agricultural Research Service (ARS), National Peanut Research Laboratory (NPRL), 1011 Forrester Dr. SE, Dawson, GA, USA

**Keywords:** Anthracnose, *Colletotrichum siamense*, Ornamental, *Camellia japonica*, Whole genome sequence

## Abstract

*Colletotrichum siamense* Prihastuti, L. Cai & K.D. Hyde is an economically important fungal pathogen that causes damage to diverse horticultural and agronomic crops including fruits, vegetables, and ornamentals. While its prevalence on, and damage to ornamental crops has been reported in many tropical and subtropical countries, to date it was not documented on ornamentals in the United States. Here, we report the isolation of *C. siamense* from *Camellia japonica* L. in the United States and the first genome sequence of this pathogen. *C. siamense* isolates GCC05 and GCC08 were obtained from *C. japonica* plants in George County, Mississippi. Whole genome sequencing of GCC05 and GCC08 was performed as paired-end reads of 150 bp using NovaSeqXPlus. Clean reads were *de novo* assembled and were also mapped to *C. siamense* reference genome sequence, Cg363, and genome-wide variants were analyzed. Sequencing data were submitted to NCBI GenBank BioProject PRJNA1273064, and accession numbers SAMN48929844 [1] and SAMN49025292 [2] were assigned to GCC05 and GCC08, respectively. The data reported here will enable the development of diagnostics towards monitoring the occurrence and spread of *C. siamense* in the United States and will contribute to developing disease management strategies. Comparative genomics and phylogenetic analyses using these genome sequences will also aid in deriving insights that add to the knowledge of diversity and pathogenicity of *C. siamense* and the *Colletotrichum gloeosporioides* species complex (CGSC).

Specifications Table

**Table:** 

Subject	Biology
Specific subject area	Fungal plant pathogen genomics
Type of data	Genome sequences, raw data, De novo assembly, filtered and trimmed reads, reference mapping data, tables, figures
Data collection	*Colletotrichum siamense* was isolated from symptomatic *Camellia japonica* plants in George County, Mississippi, USA, in 2023. The whole genome was sequenced on Illumina NovaSeqXPlus platform at the UC Davis Genome Center, California. Data were processed using CLC Genomics Workbench v25.0.1. Clean reads >120 nucleotides were *de novo* assembled, and contigs were used to generate alignments for phylogenetic identification. Clean reads were also mapped to the reference genome Cg363 and variants (SNPs, MNPs, and Indels) were identified.
Data source location	*C. siamense* was collected from George County, Mississippi, USA, 30° 51′ 54.5436′′ N, 88° 32′ 43.2054″ W. Sequencing data were placed in public repository: NCBI.
Data accessibility	*BioProject PRJNA1273064, accession numbers SAMN48929844 and SAMN49025292. Repository name: National Center for Biotechnology Information (NCBI). Direct URL to data:*https://dataview.ncbi.nlm.nih.gov/object/PRJNA1273064?reviewer=6so5e1rsfphvgqpvouimi3l034

## 1 Value of the Data

• This is the first time *Colletotrichum siamense* has been isolated from an ornamental plant, *Camellia japonica*, in the United States.
• This is the first report of whole genome sequencing of *C. siamense* on ornamental plants in the United States. Given the phenotypic plasticity of *Colletotrichum* [1], the availability of the genomes reported here will allow unambiguous identification of this pathogen in the region.
• The ornamental horticulture industry is a significant economic contributor in the specialty crops category in the United States. Identification and management of new fungal diseases is critical for reducing the damage to crops and thereby economic losses.
• The *C. siamense* genomes reported here show many genetic variants when compared to the reference genome sequence of Cg363 from China. While a large number of single- and multi-nucleotide polymorphisms (SNPs, MNPs) and Insertion/Deletions (Indels) were observed throughout the genome, SNPs were found to be the major category of variants. The genome information is critical to understand the unique and differentiating features of the isolates found in Mississippi, in the United States, compared to *C. siamense* strains predominantly reported from tropical and subtropical countries elsewhere.
• Mining and comparative analysis of *C. siamense* genome reported here and other genomes enable development of molecular markers which in turn can facilitate analysis of population structure, improve the resolution of phylogenetic trees, and identify pathogen strains to which plant cultivars can show resistance or susceptibility.
• The data reported here will be valuable for the development of diagnostics for accurate identification, tracking, and management of this pathogen on ornamentals as well as on other horticultural and agronomic crops in the United States.
• The genomic data provided here are also of substantial importance for studies on the evolution of the species and in understanding the genomic basis for broad or narrow host range of *C. siamense* pathogens.

## 2 Background

The *Colletotrichum* genus includes many important species that cause anthracnose on a wide range of host plants worldwide leading to significant economic losses, and consequently it is considered one of the top ten most damaging plant pathogens [2]. Recent diversity and phylogenomic analyses divided 280 *Colletotrichum* species into 16 species complexes and 15 singletons [3]. The *Colletotrichum gloeosporioides* species complex (CGSC) is one of the most devastating phytopathogenic species complexes and its members cause damage to diverse horticultural and agronomic crops [2,4]. *C. siamense*, a member of the CGSC, causes damage to diverse plant species including fruits, vegetables, and ornamentals in several tropical and subtropical countries [5,6].

In the United States, ornamental horticulture is a significant economic contributor, accounting for a third of the total value of specialty crops and 10 % of the value of total crop production [7]. In addition to managing existing diseases, proactively identifying, tracking, and managing new pathogens is important for maintaining the productivity of the ornamental industry. While some species of *Colletotrichum* have been reported on ornamental plants in the United States, to our knowledge, *C. siamense* has not been documented as a pathogen. Here, we provide the first report of *C. siamense* on *C. japonica*, the most important ornamental Camellia in the United States.

Accurate identification of fungal species is crucial for understanding etiology and pathogenicity, exploring host range, creating effective disease management strategies, and developing diagnostics for tracking pathogen spread [8]. Internal transcribed spacer (ITS) sequences by themselves or in combination with multi-locus concatenated sequences in phylogenetic analyses were successfully used to distinguish several *Colletotrichum* species [5]. However, even the resolution of most commonly used ITS was found to be insufficient to reliably differentiate *C. siamense* from several *Colletotrichum* species, thus requiring the use of specific genes or alternate approaches in those situations [9]. The availability of whole genome sequences of *C. siamense* and their comparison to the non-redundant reference sequences and other genomes enable differentiation of *C. siamense* from other species with high accuracy.

While *C. siamense* has been found on many hosts around the world, to date most reported cases of this species on ornamentals are from Asian countries along with reports from a few other countries that include Australia, Brazil, and Mexico. By identifying *C. siamense*, for the first time, in an ornamental crop in the United States this study extends the geographic range of *C. siamense* on ornamental crops. Most importantly, the genomic sequences provided here aid in the development of diagnostics for the identification, tracking, and management of this pathogen in the United States on horticultural as well as agronomic crops. The purpose of this research was to obtain the draft genome sequences of two *C. siamense* isolates, GCC05 and GCC08, from the United States and derive information on their similarity/dissimilarity to existing *C. siamense* genomes reported from other regions.

## 3 Data Description

**Fig. 1:**
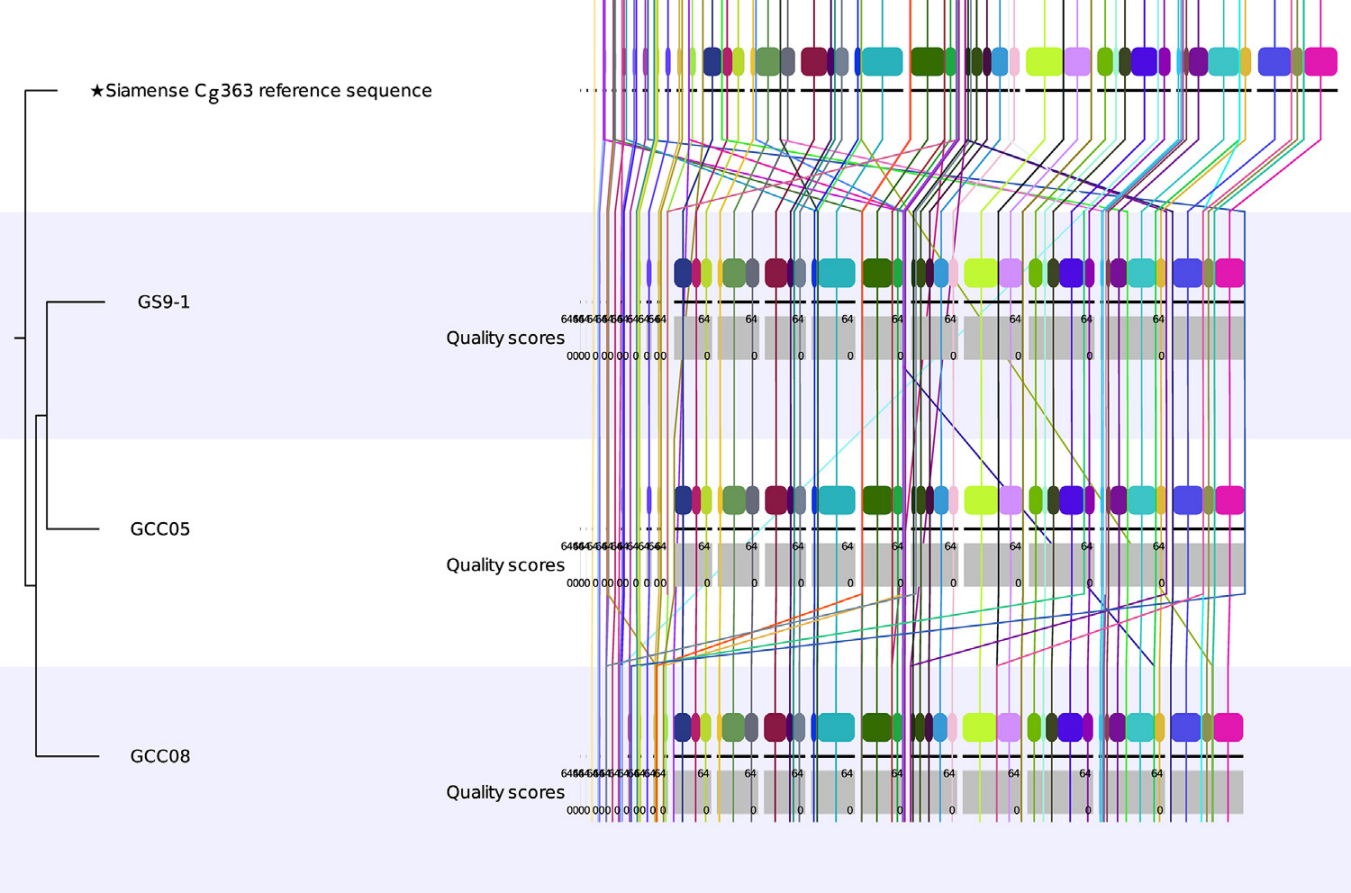
Whole genome alignment of *Colletotrichum siamense* isolates GCC05, GCC08, and GS9–1 to reference genome Cg363.

Draft genome sequences for isolates GCC05 and GCC08 are available at the National Center for Biotechnology Information (NCBI) under the BioProject Number PRJNA1273064 with the accession numbers SAMN48929844 and SAMN49025292, respectively. The summary description of sequencing data is shown in Table 1. A third isolate, GS9–1, was also sequenced and initially included in the data and analyses, but it was later determined to be genetically identical to GCC05 (Fig. 1) and was, therefore, excluded from subsequent analyses and final reporting.

**Table 1:** Genomic features and *de novo* assembly statistics for *Colletotrichum siamense* isolates GCC05 and GCC08.

Attribute	GCC05	GCC08
Genome size (bp)	57,743,335	57,651,220
Number of contigs	165	249
Largest contig (bp)	3,257,744	3,380,721
Average contig length (bp)	264,878	174,701
Sequencing Coverage	170.5x	99.9x
Number of scaffolds	116	201
N25 (bp)	2,249,227	1,275,315
N50 (bp)	1,050,410	789,599
N75 (bp)	501,940	384,783
Overall GC Content	52.37 %	52.47 %

**Fig. 2:**
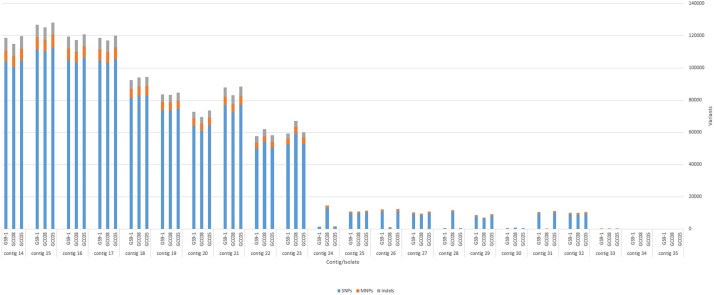
Contig-wise distribution of variants. SNPs: single nucleotide polymorphisms; MNPs: multiple nucleotide polymorphisms; and Indels: Insertions and deletions.

The whole genome sequences of the isolates were aligned to the annotated reference genome sequence of isolate Cg363 (Biosample ID: SAMN09531620) [10]. The alignment results are presented in Fig. 1. Genome-wide variant analysis was performed based on the comparison of sequencing data of the isolates and the reference genome sequence (Fig. 2). Contigs 14 to 23 shown in Fig. 2 correspond to ten core chromosomes expected in the *C. siamense* genome [11]. Additional work needs to be done to determine whether one or more of the smaller contigs merge with the larger contigs or correspond to mini-chromosomes commonly observed in several species of the CGSC, including *C. siamense* [11].

## 4 Experimental Design, Materials and Methods

### 4.1 Fungal isolation

Plant samples from *Camellia japonica* ‘Gunsmoke’ that exhibited foliar anthracnose symptoms and stem dieback were collected in George County, Mississippi, USA. Samples were surface sterilized in 10 % (v/v) sodium hypochlorite solution (1:10 dilution of commercial bleach, approx 5–9 % NaOCl), rinsed in ultra-pure sterile reverse osmosis water, and the healthy and diseased tissue margin of foliar and stem canker symptoms were placed on potato dextrose agar (PDA). Fungal isolates were recovered and pure isolates obtained by passaging mycelial tips on PDA. Resulting isolates were grown at 24 °C for 3–5 days under constant light and morphologically identified to the genus level (*Colletotrichum*) through microscopic examination of conidia and conidiophores from acervuli. Mycelial tips were extracted and grown in potato dextrose broth on a shaker for an additional 3–5 days at 24 °C in low light conditions. The resulting mycelial globules were removed and vacuum filtered to eliminate the supernatant broth. Globules were split open, and agar blocks extracted using a fire-sterilized scalpel. Globules were placed into sterile 5 ml centrifuge tubes and frozen at −80 °C overnight and subsequently placed in a freeze dryer for approximately 48 h. The lyophilized tissue was placed in a 2 ml bead beater vial approximately ¼ full of 1 mm zirconium silica beads, with 1 ml DEPC-treated, molecular biology grade water added. The vials were placed on a Biospec Products Mini-Beadbeater and beaten at 2600 rpm for 3 min at room temperature (22 ± 3 °C). (BioSpec Products, Inc, Bartlesville, OK, USA).

Isolates were deposited in the Agricultural Research Service Culture Collection at the Northern Regional Research Laboratory (NRRL) located in Peoria, IL, USA. These isolates were assigned NRRL numbers 64915 (GCC05) and 64917 (GCC08).

### 4.2 Genomic DNA preparation

DNA extraction for all three samples (GCC05, GCC08, and GS9–1) was initially performed using the Dynabeads DNA Direct Universal Kit (Fisher Scientific, USA) according to the manufacturer’s instructions; however, due to issues with impurities in GCC05, DNA was re-extracted from GCC05 using the Wizard Genomic DNA Purification Kit (Promega Corp, USA), again following the manufacturer’s instructions. The concentration of DNA was measured using a NanoDrop 1000 Spectrophotometer (Thermo Scientific, USA).

### 4.3 Genome sequencing and assembly

Microbial whole genome library (350 bp) was prepared using AB clonal Rapid Plus DNA kit. Sequencing was performed as paired-end reads of 150 base pairs using the Illumina NovaSeqXPlus platform by Novogene, at the UC Davis Genome Center (Davis, CA, USA). Genome sequencing produced a total of 43,535,962 raw reads for GCC05 and 34,337,994 raw reads for GCC08.

To generate clean reads, adapters P5 and P7 (see Table 2 for adapter sequences) were removed from the reads, and low-quality or short reads (<140 bp) were trimmed using CLC Genomics Workbench v25.0.2 (Qiagen, Aarhus, Denmark). The resultant clean reads were mapped to the reference genome of *C. siamense* (Strain ID: Cg363). *De novo* assembly was performed using the default settings in CLC Genomics Workbench v25.0.2.

**Table 2:** Adapters used by Novogene sequencing.

Adapter	Sequence
P5	AATGATACGGCGACCACCGAGATCTACAC[i5]ACACTCTTTCCCTACACGACGCTCTTCCGATCT
P7	GATCGGAAGAGCACACGTCTGAACTCCAGTCAC[i7]ATCTCGTATGCCGTCTTCTGCTTG

Genome completeness was assessed with Busco v6.0.0. Samples were compared against the Glomerellales database (glomerellales_odb12). GCC05 was assessed to have 99.3 % completeness (C:99.3 %, S:99.1 %, D:0.2 %, F:0.1 %, M:0.6 %, n:6103) while GCC08 was assessed to have 99.4 % completeness (C:99.4 %, S:99.2 %, D:0.2 %, F:0.1 %, M:0.5 %, n:6103) where *C* = complete Buscos, *S* = Single copy Buscos, *D* = Duplicate Buscos, *F* = Fragmented Buscos, and *M* = Missing Buscos.

Furthermore, genomes were scanned for contaminants using Kraken2 v2.14. Three significant contaminants were found: *Homo sapiens*, with 304 reads from GCC05 and 548 reads from GCC08; *Cutibacterium acnes* with 694 reads from GCC08 (no contamination in GCC05); and *Malassezia restricta* with 125 reads from GCC08 (no contamination in GCC05). All of these contaminant reads were removed from the raw reads before *de novo* assembly was performed.

## Limitations

Not applicable

## Ethics Statement

The current work does not involve human subjects, animal experiments, or any data collected from social media platforms.

## CRediT Author Statement

**Kenneth R. Leep:** Conceptualization**,** Methodology, Formal analysis, Data curation, Writing – original draft, Writing – review & editing; **Renee S. Arias:** Conceptualization, Methodology, Resources, Formal analysis, Writing – review & editing; **Warren E. Copes:** Conceptualization, Funding acquisition, Resources, Writing – review & editing; **Siva P. Kumpatla:** Conceptualization, Methodology, Funding acquisition, Writing – original draft, Writing – review & editing.

## Data Availability

NCBIPRJNA1273064 (Original data)
